# How to apply SHA 2011 at a subnational level in China’s practical situation: take children health expenditure as an example

**DOI:** 10.7189/jogh.08.010801

**Published:** 2018-06

**Authors:** Mingyang Li, Ang Zheng, Wenjuan Duan, Xin Mu, Chunli Liu, Yang Yang, Xin Wang

**Affiliations:** 1 Department of Orthopedics, Shengjing Hospital of China Medical University, Shenyang, China; 2 Department of Breast Surgery, The First Affiliated Hospital of China Medical University, Shenyang, China; 3 Department of Humanities and Social Sciences, China Medical University, Shenyang, China; 4 Library of China Medical University, Shenyang, China; 5 Department of Fundamental Sciences, China Medical University, Shenyang, China; * Joint first authorship

## Abstract

**Background:**

System of Health Accounts 2011 (SHA 2011) is a new health care accounts system, revised from SHA 1.0 by the Organisation for Economic Co-operation and Development (OECD), the World Health Organization (WHO) and Eurostat. It keeps the former tri-axial relationship and develops three analytical interfaces, in order to fix the existing shortcomings and make it more convenient for analysis and comparison across countries. SHA 2011 was introduced in China in 2014, and little about its application in China has been reported. This study takes children as an example to study how to apply SHA 2011 at the subnational level in the practical situation of China’s health system.

**Methods:**

Multistage random sampling method was applied and 3 532 517 samples from 252 institutions were included in the study. Official yearbooks and account reports helped the estimation of provincial data. The formula to calculate Current Health Expenditure (CHE) was introduced step-by-step. STATA 10.0 was used for statistics.

**Results:**

Under the frame of SHA 2011, the CHE for children in Liaoning was calculated as US$ 0.74 billion in 2014; 98.56% of the expenditure was spent in hospital and the allocation to primary health care institutions was insufficient. Infection, maternal and prenatal diseases cost the most in terms of Global Burden of Disease (GBD), and respiratory system diseases took the leading place in terms of International Classification of Disease Tenth Revision (ICD-10). In addition, medical income contributed most to the health financing.

**Conclusions:**

The method to apply SHA 2011 at the subnational level is feasible in China. It makes health accounts more adaptable to rapidly developing health systems and makes the financing data more readily available for analytical use. SHA 2011 is a better health expenditure accounts system to reveal the actual burden on residents and deserves further promotion in China as well as around the world.

National Health Accounts (NHA) is an internationally recognized health policy analysis and evaluation tool. It is a collection of accounts framework, indicators and methods that reflect health expenditure in all aspects, monitoring the health system financial flows [1]. Efforts to describe financial flows related to the consumption of health care can be dated back as far as the 1920s [2,3].

The system of NHA in China was set up based on the System of Health accounts 1.0 (SHA 1.0) published by the OECD in 2000. This system was made up by a tri-axial relationship accounts system, that is, health care functions (HC), health care providers (HP) and health financing schemes (HF). SHA 1.0 provides a systematic description of the financial flows. It intends to describe a health system in the perspective of expenditure. But as more countries start to implement and improve health accounts, there are increased expectations from policy makers, analysts and the general public for more information which can be gained directly through the greater amount of health expenditure data [4,5].

With the rapid development of health care, the global health system is also facing a large number of problems, for example, the complexity of health financing, the innovation of medical technology, the improvement of information systems related to health services and data availability, and the rise of population aging problems, etc. Bui [6] studied the data from NHA reports on health expenditure from 1996 and found the data were often incomplete and sometimes of questionable quality. Varied suggestions prompt the need for an update to amend some of the shortcomings apparent in SHA 1.0, as well as provide an opportunity to take some new developments into health care systems. A new international accounts instrument is required to follow the development of health system and predict future changes scientifically in certain trends, comparable both over time and across countries. With the goal of producing an international standard in health accounts, a formal collaborative effort among OECD, WHO and Eurostat was agreed upon. In 2007, the revision of the former accounts system, SHA 1.0, began with almost ten years of global experience in health accounts. Finally, in 2011, the work was accomplished and SHA 2011 was published.

Compared with SHA 1.0, SHA 2011 retains the former tri-axial relationship and develops three analytical interfaces, that is, the health care consumer interface, provision interface and financing interface. It reinforces the description of health care expenditure, namely, how it was provided and financed. SHA 2011 abandons the expression of Total Health Expenditure (THE), but recommends the usage of Current Health Expenditure (CHE), which refers to the final consumption for health care goods and services by the government, non-profit institutions and households, excluding the expenditure of fixed assets. SHA 2011 makes health accounts more adaptable to rapidly developing health systems around the world. It enhances the cross-country comparability of health care expenditure and makes the financing data easier for analytical use [7,8]. Moreover, SHA 2011 will become a more useful tool in the assessment of health systems and in the analysis of health expenditure from the perspective of consumption as a whole as the WHO plans to achieve global realization of SHA 2011 by 2017 [9].

To comply with international standards, researchers from the China National Health Development Research Center started training courses on the reform of China’s health expenditure accounts based on SHA 2011 in 2014. The results of national health expenditure accounts were reported by the Research Center in China National Health Accounts Report 2015 and some journals of the Chinese Social Sciences Citation Index (CSSCI). However, far less is known about the application of this new accounts system at the subnational level. The discussion of CHE in different age groups or diseases have not been reported yet. China is a huge country with great regional differences. It is very significant to examine how to combine the principles and methods of SHA 2011 with the concrete practice in China, both at the national and subnational level.

This study mainly analyzes how to apply SHA 2011 in Liaoning Province given the practical situation of China’s health system. As China ended its one-child policy thoroughly in 2016, a great rise in birth rate is expected. Thus, children aged from 0 to 14 were chosen as the subjects of this study. The goal of this study was to describe a detailed method to calculate CHE in the frame of SHA 2011, which can be applied to other places in China as well as other countries in the world. The results are subdivided into institutions, diseases and financing schemes associated with the tri-axial relationship, which can serve as a reference to discuss the differentiation of ages, diseases, regions and other factors on health expenditure across the nation.

## METHODS

### Data sources

The health expenditure were collected from Liaoning Health Statistical Yearbook 2015, Liaoning Health Financial Yearbook 2015, China National Health Accounts Report 2015 and Liaoning Health Accounts Report 2015. The demographic data were obtained from Liaoning statistical yearbook 2015. Most of the medical expenditure were indexed from Hospital Information System, while the others were collected from on-site investigation. STATA 10.0 was used for analysis.

### Study samples

Multistage random sampling method was applied. The first stage was choosing sample cities. Four cities – Dalian, Liaoyang, Panjin and Tieling – were chosen based on the economic development and health information management system. The second stage was at the county level; choosing one rural township and one downtown district, then choosing three township hospitals and three community-health service organization respectively. In addition, we included three villages from the township and three village clinics and individual clinics into the sample. The third stage was to choose some other public health institutions and medical institutions randomly. In total, 252 institutions were taken as study samples, including 7 provincial-level hospitals, 58 institutions in Dalian, 62 institutions in Liaoyang, 64 institutions in Panjin, 61 institutions in Tieling. The investigation included gender, age, disease, expense, medical institutions, etc. The diseases were coded with the International Classification of Disease Tenth Revision (ICD-10). Valid data of 3 532 517 samples were entered into the database after rejecting invalid or wrong messages. The range of 95% confidence interval was too small to display.

### Statistical methods

Based on the principle of SHA 2011, only the services which lead to consumption of goods are taken into the calculation of CHE. Medical income, basic expenditure subsidy and special subsidy for government-designated public health projects, which penetrate for the hospital curative services and public health services, should be included in the scope. The basic construction cost, equipment cost and depreciation expenses and other non-direct consumption of human health services, should not be taken into the counting for CHE.

In the practical situation of China’s health system, Chinese children’s health expenditure can be collected from three aspects according to the tri-axial relationship: children’s health care function (beneficiary population), service providers and financing schemes. The study takes the population under 14 years old as children, as defined by clinical medicine. The top-down accounting method is adopted, namely, to calculate the CHE of total population in 2014 in the framework of SHA2011, then assess the expenditure of children using the proportion of children under 14 years old in the survey.

#### Curative services

The fund of curative services mainly come from financial subside (basic subsidy and project subsidy) and medical income. Take the outpatient expenses of children as an example. Outpatient curative services (E_OCS_) include outpatient curative income (E_OCI_), outpatient project subsidy (E_OPS_) and outpatient basic curative expenditure subsidy (E_OBS_):

E_OCS_ = E_OCI_+E_OPS_+E_OBS_

Note that outpatient expenditure for prevention should be removed.

Curative outpatient income is calculated as the product of total outpatient income of children in Liaoning (E_OI_) and the coefficient a_i_. E_OI_ is total outpatient income in Liaoning in 2014 collected from Liaoning Health Statistical Yearbook 2015 and Liaoning Health Financial Yearbook 2015. α_i_ is the proportion of outpatient income excluding preventive expenditure in the sample institutions:

E_OCI_ = E_OI_ × α_i_

α_i_ = 1 – E_POI_/E_TOI_

E_POI_ refers to preventive outpatient income in the sample institutions. E_TOI_ refers to total outpatient income in the sample institutions.

The outpatient project subsidy consists of the curative projects arranged to the hospital, including central and local subsidy policies and regulations. The data are taken from the central government subsidy, local health special funds statistics and the government health monitor system.

The calculation of outpatient basic curative expenditure subsidy is partly similar to E_OCI_. First collect basic expenditure subsidy (E_BS_) and calculate the coefficientα_s_ based on the equivalent person allocation principle. The product of E_BS_ andα_s_ is basic curative expenditure subsidy (E_BCS_). Then, introduce a new coefficient β to share E_BCS_ into outpatient basic curative expenditure subsidy (E_OBS_) according to the equivalent workload between inpatient and outpatient services.

E_BCS_ = E_BS_ × α_s_

E_OBS_ = E_BCS_ × (1 – β)

α_s_ = N_MS_/(N_MS_+N_PHS_)

β = N_IBD_/(N_IBD_+N_IOV_ × Κ) × Κ = 0.1

N_MS_ is the number of children patients treated by medical services during the survey. N_PHS_ is the number joining in the public health services. N_IBD_ is inpatient bed days. N_OV_ is outpatient visits.

Note that there are some outpatient visits for preventive services, which should be removed:

N_IOV_ = N_OV_ × Υ

Υ = 1 – N_POV_/N_TOV_

N_OV_ is total outpatient visits in Liaoning in 2014 collected from Liaoning Health Statistical Yearbook 2015 and Liaoning statistical yearbook 2015. N_POV_ refers to outpatient visits for preventive services in the survey. N_TOV_ refers to total outpatient visits in the survey.

The calculation of inpatient subsidy is similar to the outpatient subsidy. A little difference is the inpatient bed days need not exclude preventive services.

To differentiate the source of the fund, the curative service expenditure can be divided into different financing scheme. The basic curative expenditure subsidy and other government project subsidy should be brought into government financing scheme, while social insurance, commercial insurance and other public donations should be brought into social financing scheme. The rest are assigned to family financing scheme.

#### Other expenditure

The method to calculate prevention expenditure is based on the comparison between income and expense of each prevention project. If income < expense, take service expense to calculate, otherwise take service expense. Assistant services, medicine cost, management cost should also be brought into accounts. Then divide each item into government, social or family financing scheme.

## RESULTS

### Basic result of CHE for children

The CHE for children aged 14 years old and below was US$ 0.74 billion (CNY 4.573 billion, average US$1 = 6.20 CNY in 2014), accounting for 6.19% of total population. Per capita CHE for children was US$ 165.04, compared to US$ 716.82 for over 65 years old and US$ 228.16 for the rest. The infants below 1 year of age account for most of the expenditure, about 42.08%. The proportion kept decreasing with age between 1 and 11 years old, from 9.53% to 1.79%. From 11 to 14 years, the proportion varied from 2.30% to 2.37%.

### Allocation of CHE in different medical institutions

**Table 1** shows that most of CHE for children were spent in hospital, USD 0.73 billion, which accounted for 98.56% of CHE. By contrast, the expenditure in primary health agents were only USD 2.18 million. In primary health agents and outpatient service institutions, outpatient cost is more than inpatient cost. However, conversely, inpatient cost predominated in maternal and child-care service centers and hospitals, except in specialized hospitals. Among different hospitals, general hospitals took over 95% outpatient expenditure and 99% inpatient expenditure.

**Table 1:** Allocation of Current Health Expenditure (CHE) in different medical institutions

	Outpatient (US$ million)	Inpatient (US$ million)	Total (US$ million)
Primary health agents	1.70	0.48	2.18
Outpatient service institutions	3.40	0	3.40
Maternal and child care centers	0.31	4.72	5.03
Hospitals:	30.82	696.14	726.96
general hospital	29.38	694.09	723.47
specialized hospital	0.07	0.03	0.10
traditional Chinese medical hospital	1.37	2.02	3.39

### Allocation of CHE in terms of diseases and ages

CHE can be classified to reveal the cost and financing conditions of different diseases. Subdivision by age can reflect the health burden of different age groups and provides evidence for primary prevention.

**Figure 1:**
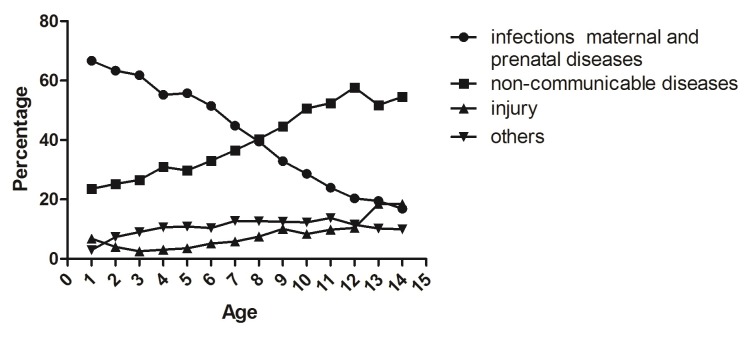
Allocation of Current Health Expenditure (CHE) for children in terms of Global Burden of Disease (GBD).

In terms of GBD, the most expenditure was spent on infections, maternal and prenatal diseases (49.88%). However, as age rises (**Figure 1****)**, the proportion of infections, maternal and prenatal diseases dropped off gradually. By contrast, non-communicable diseases and injury kept rising year-on-year. The proportion of non-communicable diseases outnumbered 50% since 10 years old.

In terms of ICD-10 (**Table 2**), respiratory system diseases accounted for the most expenditure, 43.07%, which is far above that of other diseases. The second were symptoms, signs, clinical and laboratory abnormalities, which meant the definite diagnosis of children diseases was not easy to make. After subdivision by age, respiratory system diseases still took the leading place, but decreased with age. Under one year of age, the proportion of perinatal diseases reached 22.17%, which deserves special attention. Tumors, blood diseases, endocrine, nutritional and metabolic disease, mental and behavioral disorders, digestive system diseases, skin and subcutaneous tissue disease, muscular and connective tissue diseases and urogenital system diseases kept rising for 10-year-olds overall. Nervous system diseases, eye diseases, ear diseases and chromosomal abnormalities and injury, poisoning and external causes declined before five years of age, and then kept rising. In total, most diseases showed changes before 5 years of age, especially within the first year.

**Table 2:** Allocation of CHE for 0-14-y-old children in terms of ICD-10

	0-14	0-1	1-2	2-3	3-4	4-5	5-6	6-7	7-8	8-9	9-10	10-11	11-12	12-13	13-14
Infectious diseases and parasitic diseases	3.92	6.67	7.51	7.44	7.37	8.82	7.71	6.03	4.69	4.01	3.64	2.73	2.41	2.25	4.27
Tumor	1.43	1.20	1.66	1.96	1.93	1.93	3.32	2.84	3.42	4.74	3.81	4.98	5.46	8.13	6.61
Blood diseases	0.90	0.84	1.65	0.91	1.91	1.80	2.28	3.28	3.84	4.13	3.33	4.45	4.18	3.26	4.45
Endocrine, nutritional and metabolic disease	6.12	0.85	0.69	1.31	1.11	0.60	0.63	1.10	1.25	2.45	2.88	3.99	5.48	3.34	3.97
Mental and behavior disorders	0.26	0.07	0.08	0.07	0.12	0.28	0.39	0.69	0.99	1.71	1.56	1.72	1.55	1.01	1.17
Nervous system diseases	0.99	3.15	3.57	3.23	4.39	3.68	4.85	5.56	6.06	7.03	7.44	8.47	8.21	6.65	5.91
Eye diseases	1.90	0.37	0.60	0.45	0.40	0.49	0.63	0.72	0.70	0.84	0.76	0.65	0.74	0.61	0.60
Ear diseases	0.95	0.09	0.33	0.73	0.83	0.66	0.69	0.90	0.64	0.68	0.48	0.41	0.59	0.50	0.50
Circulatory system diseases	6.88	2.52	0.90	1.43	0.88	1.19	2.27	1.61	1.54	2.37	4.64	2.79	1.85	2.78	2.68
Respiratory system diseases	43.07	37.87	56.97	59.62	56.38	57.24	52.48	47.67	43.10	34.39	31.77	26.75	23.96	21.72	17.40
Digestive system diseases	5.72	5.84	5.77	5.08	4.67	3.31	3.35	4.96	5.90	7.28	6.78	8.61	10.79	9.25	10.18
Skin and subcutaneous tissue disease	5.48	0.39	1.60	1.94	2.04	1.36	1.26	1.56	1.80	1.62	2.58	1.55	2.13	1.16	1.75
Muscular and connective tissue diseases	0.39	0.99	1.34	0.93	0.95	1.02	1.06	1.06	1.26	1.53	1.33	1.96	2.42	1.74	4.11
Urogenital system diseases	1.37	1.53	1.95	1.56	1.83	2.05	1.95	2.08	3.24	4.37	5.33	7.37	5.36	8.13	4.53
Pregnancy, childbirth and puerperium diseases	0.48	0.18	0.03	0.02	0.01	0.01	0.02	0.03	0.01	0.00	0.01	0.01	0.00	0.00	0.01
Perinatal diseases	2.80	22.17	0.94	0.20	0.02	0.01	0.00	0.01	0.02	0.02	0.02	0.01	0.03	0.02	0.01
Congenital anomalies and chromosomal abnormalities	0.21	5.50	2.96	1.57	1.40	1.06	1.62	1.34	1.36	1.38	1.47	1.87	1.61	2.49	1.59
Symptoms, signs, clinical and laboratory abnormalities	9.29	2.42	6.68	8.35	9.75	10.22	9.71	11.27	11.45	10.50	10.45	9.11	10.84	6.76	8.20
Injury, poisoning and external causes	3.21	6.80	4.05	2.53	3.09	3.55	5.08	5.77	7.41	9.11	8.02	10.29	10.90	17.31	19.78
Death	0.05	0.00	0.01	0.01	0.02	0.03	0.07	0.04	0.06	0.21	0.13	0.02	0.12	0.03	0.13
Factors influencing health status	4.59	0.56	0.72	0.66	0.89	0.69	0.63	1.47	1.24	1.62	3.58	2.28	1.36	2.86	2.15

CHE – child health expenditure, ICD 10 – International Classification of Disease, 10^th^ Revision

### Health financing schemes

As shown in **Table 3**, medical income, which mainly came from curative inpatient income and outpatient income, contributed most to health financing. In financial subsidies, basic subsidy was greater than project subsidy. The primary medical institutions could get extra subsidy from superior institutions, while some scientific hospitals and teaching hospitals had extra science and education income. But both contributed little to health financing.

**Table 3:** Health funding schemes from different institutions

Health financing	US$ (million)
Financial subsidy:	27.05
basic subsidy	19.32
project subsidy	7.73
Superior institution subsidy (only primary medical institutions)	0.13
Medical income	591.40
Science and education income (only hospitals)	0.02
Others	7.73
Total	626.33

## DISCUSSION

To our knowledge, this study is the first one to give a comprehensive report about the implementation of SHA 2011 in China at the subnational level. The study introduced the method to calculate CHE in the framework of SHA 2011. According to the needs of health financing analysis, SHA 2011 increases the benefit dimension in recurrent health expenditure, which can be sub-divided into diseases, age, sex, income and regions.

Compared with the former method of THE, CHE can monitor the disease economic burden more accurately [10]. CHE reflects the residents’ health service consumption, excluding the investment of fixed assets, such as medical instruments, construction of infrastructure and establishment of databases. According to Zhang’s study [11], in 2012 family health expenditure reached 42.60% of CHE, but at the same time personal health expenditure constituted only 34.34% of THE. The data collected from China Health Yearbook also showed that, from 1988 to 2013, personal health expenditure was always at a high level, with the peak even reaching 60%. Though the proportion of personal health expenditure in THE has declined rapidly since 2011, the residents’ feeling of it being “more expensive to see a doctor” was even stronger, showing that actual disease economic burden was heavier than what THE showed [12]. A survey indicates about 7% of the population in China fall to poverty due to personal health expenditure annually [13]. Therefore, it is better to use CHE to reveal the actual burden on residents.

**Figure 2:**
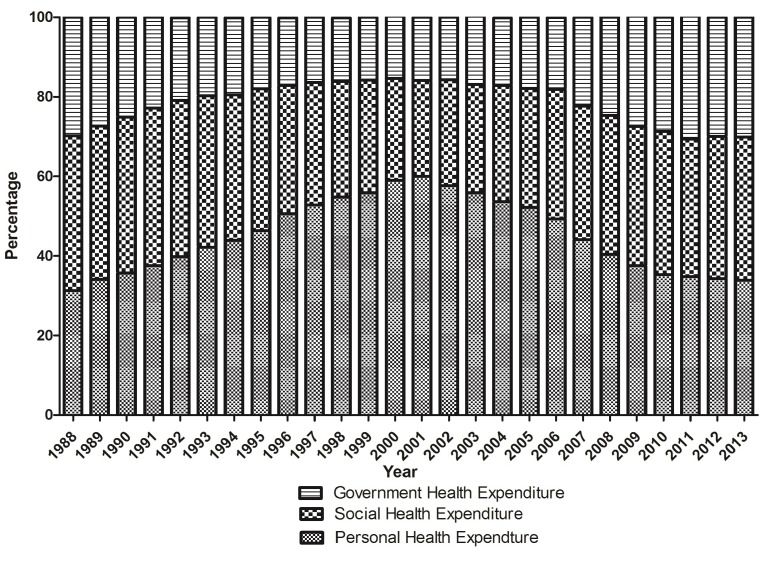
The constitution of total health expenditure.

The former data of THE also showed a heavy personal health economic burden (**Figure 2**). It also found that the government contributed the least to THE, even dropping to 15.47% in 2000. Though after that the ratio rose gradually and reached 30.1% in 2013, the practical need has not yet been matched. It is believed that reductions in government health expenditure are associated with significant rises in child mortality [14]. WHO introduces “the general government expenditure on health as a percentage of total expenditure on health” to discuss health financing. China was at 55.79% in 2014, lower than Canada (70.93%), Germany (76.99%) and Australia (67.04%) [15]. As shown in **Figure 2**, social health expenditure is almost stable, while personal health expenditure has a negative correlation to government health expenditure. Therefore, the government needs to claim more responsibility to reduce people’s disease economic burden.

Wan’s study [16] showed that the proportion of family health expenditure in children health expenditure (55.16%) was significantly higher than that of the national average level (42.60%). Meanwhile, the proportion of social health insurance program in children’s health expenditure (14.02%) is obviously lower than that in national CHE (31.84%). This is because what most children participate in is Basic Medical Insurance and New Rural Cooperative Medical Insurance, for which the compensation is lower than in Urban Employee Medical Insurance. At the same time, the proportion of outpatient expenses of children is higher than the inpatient expenses, but the compensation of the outpatient expenses is far lower, which leads to a higher proportion of the family health expenses in the children’s health expenses. To improve the medical insurance policy in the future, more medical insurance compensation policy fit for children should be set up. Outpatients should be given compensation for common or high-cost diseases.

In 2016, China ended its one-child policy. The birth rate is expected to keep rising for years, together with the increase of diseases. Therefore, the targeted prevention towards children common diseases is of great importance. This study revealed the diseases with high consumption of health resources, showing that infection, maternal, and prenatal diseases cost most in terms of GBD, and the respiratory system diseases took the leading place in each age group in terms of ICD-10, similar to the study from the Lancet [17]. Guo and his partners [18] studied the national expenditure on treatment for children, and reached the same conclusion in terms of both GBD and ICD-10. They also found that preventive service cost constituted about 20% of total health expenditure, but the proportion of government financing was less than 50%, indicating that most of the burden was shifted to households and health institutions. Liao’s study [19] also emphasized the frequency of respiratory diseases in children and suggested the prevention should be started from the most common and frequently occurring diseases. A reasonable direction of government investment would be towards diseases with high risks or heavy burden.

It is noteworthy that children grow up fast and diseases clearly vary with the rise of age. It is necessary to subdivide children into several age groups to reveal the differences in each group and make it more accurate and convenient introduce prevention and medical treatment. **Table 2** shows the large changes in disease spectrum at different ages. Bui’s study [20] in the US indicated that the cost per child was greatest for infants younger than 1 year old. As for total health expenditure, Cidav [21] reported that expenditure increased by 5% with each year of age, from 3 years of age. The study also found the expenditure for long-term care, case management, partial hospitalization, medication management and respite treatment increased with age, and declined for physical therapy, mental health services, assessment services and family therapy. Therefore, according to the seriousness of different diseases, these should be strengthened pertinently to cut down the morbidity of infectious diseases and perfect prenatal screening and diagnosis. The differences of burden provide a study basis on health expenditure for future demographic changes.

The curative care expenditure amounted for 83.08% of CHE in Liaoning, higher than the national level (75.59%) reported by Chai and her partners [22]. They also compared the number to the other countries, indicating that the proportion in China was much higher than that in Germany (67.07%), Canada (66.32%), France (65.97%) and Korea (68.68%), which suggested that China’s health resources were more extensively allocated to treatment services. Moreover, 44.20% of treatment expenditure was expended by the family. However, in OECD countries it accounted for only 11.15%. If the health care system is over-reliant on family health expenditures, it can easily lead to catastrophic expenditure and poverty. As China’s medical insurance consists of basically universal coverage, it seems that the burden on household should have been relieved. In reality, the reimbursement rate is lower than required, and the insurance details still need to improve based on the practical situation of different districts. Berdahl’s study [23] in the United States indicated that children’s health access, utilization and expenditure improved significantly with the spread of insurance coverage, but racial, income and insurance disparities were the negative factors. China is the most populous country, so the regional and income differences deserve close attention during study and policy-making.

In addition, China has been pushing its “hierarchical medical system” in recent years, aiming at providing first-line treatment at primary health care institutions, such as family doctors, community-health service centers or township health clinics, if necessary, and then transferring to hospitals. Our study found that 63.61% of medical funds were used in hospitals, while the proportion in primary health care institutions was lower than 20%, not achieving expectations. Cohen’s study [24] found that the top 1% most expensive children patients accounted for one-third of pediatric health spending, and hospital care accounted for nearly 80% of the spending for those children. Despite this finding, 40% of children patients with complex chronic diseases may not achieve an annual primary care visit [25]. Therefore, many problems exist in the financial flow of medical treatment between hospitals and primary health care institutions. The hierarchical medical system should be strengthened further.

The strength of this study is that 3 532 517 samples were involved, and that the new account method to calculate CHE in the frame of SHA 2011 is reported for the first time. There are also some limitations. Only the accounts result of CHE in 2014 have been included. There was no comparison with CHE in earlier years or in other provinces, as few related studies have been published. Some potential influencing factors of CHE, such as gender or length of bed days, are not discussed either. Further studies with cross-national data would do well to discuss these factors.

## CONCLUSIONS

SHA 2011 is a better health expenditure accounts system to reveal the actual burden on residents. The method of applying SHA 2011 at the subnational level is feasible in China. It makes health accounts more adaptable to rapidly developing health systems and makes the financing data more readily available for analytical use. Under the frame of SHA 2011, the CHE for children in Liaoning was calculated as US$ 0.74 billion in 2014. Most of the expenditure was spent in hospitals, and the proportion of outpatient expenses of children was higher than the inpatient expenses. Infection, maternal and prenatal diseases cost most in terms of GBD, and respiratory system diseases were in the leading place in terms of ICD-10. In addition, Medical income contributed most to the health financing. Most of the funds were allocated to treatment services, and the allocation to primary health care institutions was insufficient. In the light of that, further promotion of SHA 2011 and improvement of government investment, redistribution of the allocation system and reform of the health care system should attract wide attention.
